# The Childhood Acute Illness and Nutrition (CHAIN) network nested case-cohort study protocol: a multi-omics approach to understanding mortality among children in sub-Saharan Africa and South Asia

**DOI:** 10.12688/gatesopenres.13635.1

**Published:** 2022-07-18

**Authors:** James M. Njunge, Kirkby Tickell, Abdoulaye Hama Diallo, Abu Sadat Mohammad Sayeem Bin Shahid, Md. Amran Gazi, Ali Saleem, Zaubina Kazi, Syed Ali, Caroline Tigoi, Ezekiel Mupere, Christina L. Lancioni, Emily Yoshioka, Mohammod Jobayer Chisti, Moses Mburu, Moses Ngari, Narshion Ngao, Bonface Gichuki, Elisha Omer, Wilson Gumbi, Benson Singa, Robert Bandsma, Tahmeed Ahmed, Wieger Voskuijl, Thomas N. Williams, Alex Macharia, Johnstone Makale, Anna Mitchel, Jessica Williams, Joe Gogain, Nebojsa Janjic, Rupasri Mandal, David S. Wishart, Hang Wu, Lei Xia, Michael Routledge, Yun Yun Gong, Camilo Espinosa, Nima Aghaeepour, Jie Liu, Eric Houpt, Trevor D. Lawley, Hilary Browne, Yan Shao, Doreen Rwigi, Kevin Kariuki, Timothy Kaburu, Holm H. Uhlig, Lisa Gartner, Kelsey Jones, Albert Koulman, Judd Walson, James Berkley

**Affiliations:** 1 The Childhood Acute Illness and Nutrition Network, KEMRI/Wellcome Trust Research Programme, Nairobi, Kenya; 2 KEMRI-Wellcome Trust Research Programme, Kilifi, Kenya; 3 Global Health and Epidemiology, University of Washington, Seattle, Seattle, USA; 4 Department of Public Health, Faculty of Health Sciences, University of Ouagadougou, Ouagadougou, Burkina Faso; 5 Nutrition and Clinical Services Division, International Centre for Diarrhoeal Disease Research, Bangladesh (icddr,b), Dhaka, Bangladesh; 6 Department of Pediatrics and Child Health, Aga Khan University Hospital, Karachi, Karachi, Pakistan; 7 Department of Paediatrics and Child Health, College of Health Sciences, Makerere University, Kampala, Uganda; 8 Department of Pediatrics, Oregon Health and Science University, Portland, OR, USA; 9 Kenya Medical Research Institute, Nairobi, Kenya; 10 Centre for Global Child Health, The Hospital for Sick Children, Toronto, Ontario, Canada; 11 Department of Biomedical Sciences, University of Malawi College of Medicine, Blantyre, Malawi; 12 Amsterdam UMC location, University of Amsterdam, Amsterdam, The Netherlands; 13 Amsterdam Centre for Global Child Health & Emma Children’s Hospital, Amsterdam, The Netherlands; 14 Institute of Global Health Innovation, Department of Surgery and Cancer, Imperial College London, London, UK; 15 SomaLogic, Boulder, Colorado, USA; 16 Department of Biological Sciences, University of Alberta, Edmonton, Alberta, Canada; 17 School of Food Science and Nutrition, University of Leeds, Leeds, UK; 18 School of Food and Biological Engineering, Jiangsu University, Zhenjiang, China; 19 Departments of Anesthesiology, Pain, and Perioperative Medicine, Stanford University School of Medicine, Stanford, CA, 94305, USA; 20 Department of Pediatrics, Stanford University School of Medicine, Stanford, CA, 94305, USA; 21 Department of Biomedical Data Science, Stanford University School of Medicine, Stanford University School of Medicine, Stanford, CA, 94305, USA; 22 Division of Infectious Diseases and International Health, University of Virginia, Charlottesville, Virginia, USA; 23 Wellcome Sanger Institute, Hinxton, UK; 24 The Centre for Microbiology Research, Kenya Medical Research Institute, Nairobi, Kenya; 25 Translational Gastroenterology Unit, John Radcliffe Hospital, University of Oxford, Oxford, UK; 26 Department of Paediatrics and Biomedical Research Centre, University of Oxford, Oxford, UK; 27 Kennedy Institute of Rheumatology, University of Oxford, Oxford, UK; 28 Gastroenterology Department, Great Ormond Street Hospital for Children, London, UK; 29 MRC Epidemiology Unit, University of Cambridge, Cambridge, UK; 30 NIHR BRC Nutritional Biomarker Laboratory, University of Cambridge, Cambridge, UK; 31 Center for Tropical Medicine and Global Health, University of Oxford, Oxford, UK

**Keywords:** Case-Cohort, Mortality, Children, LMIC, Omics, Systems Biology

## Abstract

**Introduction**: Many acutely ill children in low- and middle-income settings have a high risk of mortality both during and after hospitalisation despite guideline-based care. Understanding the biological mechanisms underpinning mortality may suggest optimal pathways to target for interventions to further reduce mortality. The Childhood Acute Illness and Nutrition (CHAIN) Network ( www.chainnnetwork.org) Nested Case-Cohort Study (CNCC) aims to investigate biological mechanisms leading to inpatient and post-discharge mortality through an integrated multi-omic approach.

**Methods and analysis**; The CNCC comprises a subset of participants from the CHAIN cohort (1278/3101 hospitalised participants, including 350 children who died and 658 survivors, and 270/1140 well community children of similar age and household location) from nine sites in six countries across sub-Saharan Africa and South Asia. Systemic proteome, metabolome, lipidome, lipopolysaccharides, haemoglobin variants, toxins, pathogens, intestinal microbiome and biomarkers of enteropathy will be determined. Computational systems biology analysis will include machine learning and multivariate predictive modelling with stacked generalization approaches accounting for the different characteristics of each biological modality. This systems approach is anticipated to yield mechanistic insights, show interactions and behaviours of the components of biological entities, and help develop interventions to reduce mortality among acutely ill children.

**Ethics and dissemination**. The CHAIN Network cohort and CNCC was approved by institutional review boards of all partner sites. Results will be published in open access, peer reviewed scientific journals and presented to academic and policy stakeholders. Data will be made publicly available, including uploading to recognised omics databases.

**Trial registration** NCT03208725.

## Introduction

In 2019, an estimated 5.2 million children under five years died worldwide, predominantly in sub-Saharan and South Asia and mainly due to infectious causes ^1, 2^. When children in low- and middle-income countries (LMIC) experience an acute illness, their average mortality risk during and after a hospitalisation event remains unacceptably high and can exceed 20% ^3– 11^. This high mortality risk may be due to illness severity, underlying comorbidities such as malnutrition, limited healthcare access and resources, and poverty. The results of the CHAIN cohort, as well as other studies, suggest that half of all mortality in children admitted for acute illness in these settings occurs after discharge ^5, 11, 12^ but a large group of children can be identified as low risk. In the past decade, very few novel biological interventions have demonstrated the ability to reduce mortality among children in LMIC with acute illnesses ^13, 14^. Interventions that have been tested in clinical trials have often been translated from high-income settings rather than being based on a thorough mechanistic understanding of child mortality in LMICs. Finally, typical interventions commonly do not address the multiple biological factors that contribute to acute and post-discharge mortality such as the inciting illness, nutrient deficiencies and other comorbidities including enteropathy and intestinal dysbiosis ( Box 1). These observations highlight the need for an improved understanding of the mechanisms leading to death and for the development of new interventions for prevention of morbidity and mortality.

The Childhood Acute Illness and Nutrition (CHAIN) Network aims to improve child survival by improving understanding and identifying intervenable mechanisms leading to death among acutely ill children in sub-Africa and South Asia ^58^. The CHAIN cohort was a prospective observational study at nine hospitals which enrolled children aged two to 23 months admitted with acute illness, stratified by anthropometric status, to investigate mortality despite guideline-based care associated with their admission event and for 6 months after discharge. This protocol describes the CHAIN Nested Case-Cohort (CNCC) study that brings together a consortium of laboratories to efficiently utilise systematically collected samples from the CHAIN cohort to examine biological pathways leading to mortality. This analysis will be both targeted based on prior literature and untargeted discovery work that will generate new hypotheses. Systemic proteome, metabolome, intestinal microbiome, lipidome, lipopolysaccharides, haemoglobin variants, toxins, pathogens and biomarkers of enteropathy will be determined. Computational systems biology analyses across these meta-dimensional data modalities are expected to deliver more comprehensive mechanistic insights into the biological pathways and converging pathological processes leading to mortality. These data will inform the development of novel interventions aimed at mortality risk reduction among acutely ill children.

**Box 1. Multiple and Interconnected biological pathways lead to mortality**

***Infection***

Acutely ill children in LMICs typically present with respiratory infection, diarrhoea or encephalopathy and across these syndromes, those with undernutrition are at higher risk of mortality. While deaths are typically associated with these infectious syndromes, positive bacterial blood cultures are found in only <10% of cases ^15, 16^. Studies including The Global Enteric Multicenter Study (GEMs) and The Pneumonia Etiology Research for Child Health (PERCH) have identified epidemiological associations between molecular evidence of specific bacterial and viral pathogens and mortality ^17, 18^. However, the pathways to mortality during such infections, other than via overwhelming sepsis, are unclear.

***Systemic inflammation and altered metabolism***

Mortality has been associated with a sepsis-like host-response profile characterized by systemic inflammation and altered metabolism linked to bioenergetic deficits ^2, 19– 22^. In sepsis, mortality has been linked to immunoparalysis, organ damage including the cardiac, respiratory, hepatic and renal systems, uncontrolled inflammation, proteolysis, and defects of organ healing capability ^23– 34^. Mitochondrial and peroxisomal dysfunction involves kynurenine derivatives including intermediates of redox cofactors nicotinamide adenine dinucleotide (NAD), acylglycerophosphocholines, acyl-carnitines, and bile acids. There is also a well-recognised association between the degree of mitochondrial dysfunction and sepsis outcomes suggesting a role of immunometabolism and suggesting new routes for therapeutic intervention ^35– 37^. There is much overlap in mechanisms described in sepsis and in severely malnourished children suggesting that deaths in malnutrition are largely due to sepsis and that malnutrition may predispose to these phenomena when sepsis occurs ^23^. Finally, the role of hormones in metabolic regulation and reprogramming in sepsis and undernutrition has not been well investigated but recent studies show that leptin is associated with mortality ^19, 20, 38^.

***Nutrient deficiencies***

There is limited understanding of micronutrient pathophysiology and requirements during illness especially among children in LMIC. Micronutrient deficiencies are known underlying contributors to increased risk of morbidity and mortality with iron, iodine, folate, vitamin A, and zinc deficiencies being the most widespread ^39, 40^. During critical illness, micronutrient deficiency may impair enzyme activity and vital processes such as energy metabolism and contribute to oxidative stress ^40, 41^. Disrupted energy supply to cellular processes and organ dysfunction may result in final common pathways leading to mortality, or syndrome-specific pathways such as energetic exhaustion due to a prolonged, greatly increased work of breathing in pneumonia. Previous studies have mostly not investigated the mortality occurring in children soon after hospital discharge and prolonged elevated mortality risk, although long-term immune paralysis, metabolic and functional abnormalities are recognised phenomenon post-sepsis or ICU care ^42– 44^.

***Enteric dysfunction***

It is recognised that many children in LMIC settings often exhibit intestinal mucosal changes, including inflammation, epithelial barrier dysfunction (leaky gut) and blunted villi which are similar to those observed in children with environmental enteric dysfunction ^45– 47^. Enteric barrier dysfunction may lead to translocation of organisms into the circulation. A circulating microbiome consistent with intestinal microbiota ( *Lactobacillus sp., Bacteroides sp., and Streptococcus sp*.) has been correlated with products of gut bacterial translocation such as lipopolysaccharide (LPS) and D-lactate and systemic inflammation and predicted adverse cardiovascular events ^48^. Biomarkers of enteric dysfunction ^49^ have not been assessed in relation to mortality risk or linked to specific infection episodes. However, limited data from LMIC settings suggests that in children with severe malnutrition, intestinal inflammation is linked to mortality via systemic inflammation rather than directly ^50^.

***The gut microbiota***

The gut microbiota protects against pathogens and maintains normal immune and metabolic homeostasis ^51– 53^. Loss of diversity and imbalance in composition of the microbiota, termed dysbiosis, is clearly described but associations with mortality risks are not. Dysbiosis is likely correlated with illness severity and has been linked to increased mortality in critically ill patients including sepsis ^54, 55^, in patients undergoing allogeneic hematopoietic-cell transplantation ^56^, in hospitalized patients with cirrhosis ^57^ among others. A marked insult on the microbiome is likely when children are hospitalized and exposed to nosocomial microorganisms, and to selection pressure from antimicrobials, resulting in acquisition of antimicrobial resistance genes. The health consequences of these likely hospital-acquired organisms, which may also differ in virulence from community-derived species, is unclear.

***Host genetics and toxins***

Other contributors to morbidity and mortality among acutely ill children in LMIC may include pre-existing conditions such as sickle cell disease and exposure to toxins including aflatoxins.

## Methods

**Ethical approval:** The study protocol was reviewed and approved by the Oxford Tropical Research Ethics Committee, UK; the Kenya Medical Research Institute, Kenya; the University of Washington and Oregon Health and Science University, USA; Makerere University School of Biomedical Sciences Research Ethics Committee and The Uganda National Council for Science and Technology, Uganda; Aga Khan University, Pakistan; the International Centre for Diarrhoeal Disease Research, Bangladesh; The University of Malawi; The University of Ouagadougou and Centre Muraz, Burkina Faso; the Hospital for Sick Children, Canada; and University of Amsterdam, The Netherlands.

### Participant enrolment and data collection

The objectives of the CHAIN cohort study, participating sites, patient and public involvement, eligibility, screening, enrolment and follow-up procedures, and specimen collection have been detailed previously ^58^. Briefly, 3101 acutely ill children were recruited from nine sites in six countries in sub-Saharan Africa and South Asia. Children were enrolled at admission to hospital and followed up for 180 days after discharge from hospital. Participating sites included rural and urban hospitals in Bangladesh (icddr,b Dhaka Hospital and Matlab Hospital), Burkina Faso (Banfora Regional Referral Hospital), Kenya (Kilifi County Hospital, Mbagathi Sub-County Hospital and Migori County Referral Hospital), Malawi (Queen Elizabeth Central Hospital), Pakistan (Civil Hospital, Karachi), and Uganda (Mulago Hospital). The mid-upper arm circumference (MUAC) was used to enrol children into three strata that included not wasted (MUAC ≥12.5cm for age ≥six months or MUAC ≥12cm for age <six months), moderately wasted (MUAC 11.5 to <12.5 cm for age ≥six months or MUAC 11 to <12 cm for age <six months), and severely wasted or kwashiorkor (oedematous malnutrition) (MUAC <11.5cm for age ≥six months or MUAC <11cm for age <six months or bilateral pedal oedema unexplained by other medical causes). In addition, children in the same communities as hospitalised cases who were not acutely ill: community participants (CP, N=1140) were recruited at a single time-point to establish community norms for demographic and biological factors.

Samples collected at admission and discharge from hospitalised children and from community participants will be utilised in the CNCC together with characteristics of study participants: demographic (age, sex, and site), clinical (shock, severe inflammatory response syndrome, consciousness level, pneumonia, diarrhoea, malaria, HIV, anaemia, blood glucose), anthropometric (nutritional oedema/kwashiorkor, MUAC, and z scores for weight-for-length, weight-for-age, and length-for-age), social data (caregiver characteristics, household-level exposures, and access to health care) and other laboratory tests (clinical chemistry, complete blood count, rectal swab and blood culture) that were systematically undertaken in real-time at each site. Case report forms used to collect data and clinical and sample handling SOPs can be downloaded at www.chainnetwork.org/resources.

### Samples collection, processing, and storage

**Figure 1.**
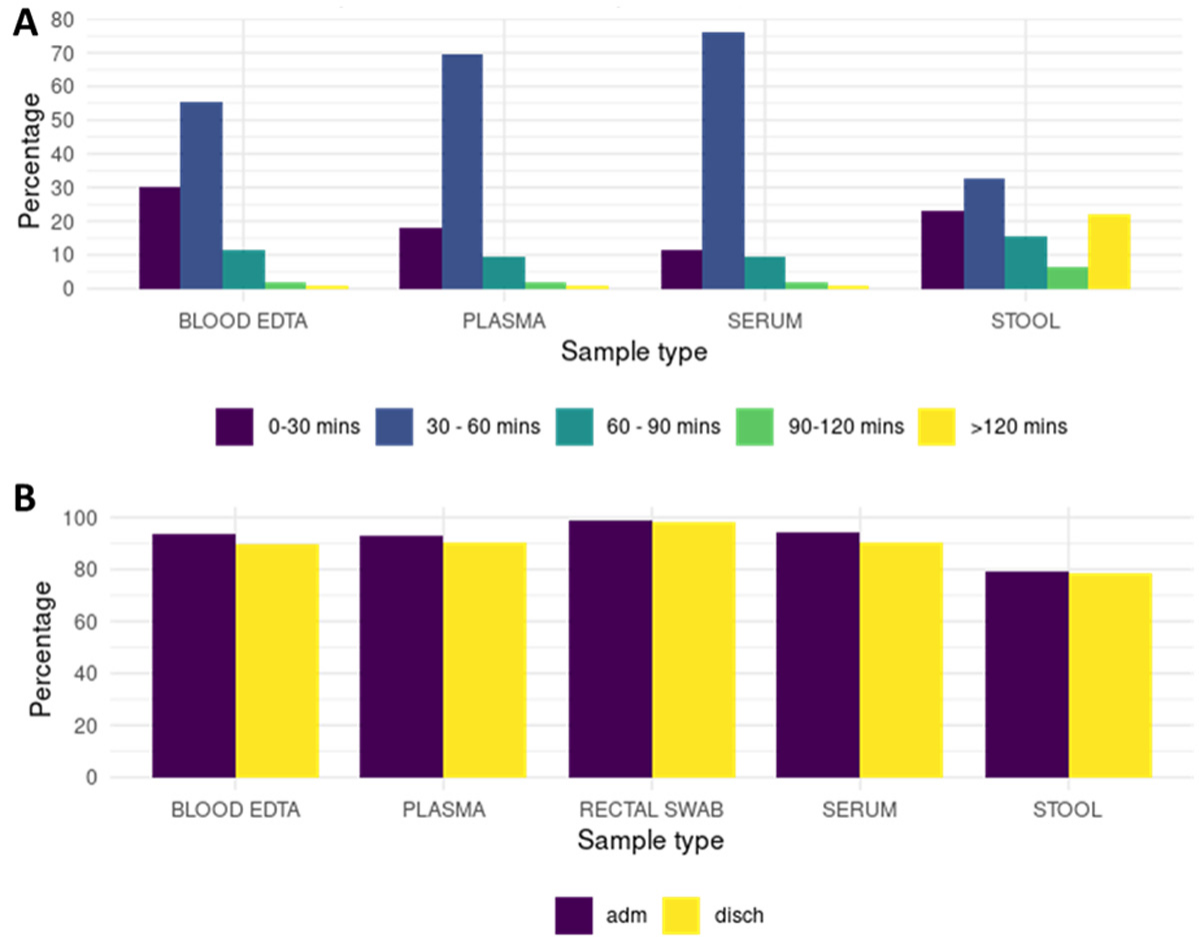
**A.** Duration of time from sample collection to storage at -80°C. ~80% of the samples (whole blood, plasma, serum, and rectal swabs) achieved a median of 44 minutes from collection to storage at a -80°C freezer. Except for stool, the rest of the samples were frozen at a -80°C freezer within 1 hour of collection. **B.** Proportion of samples available for analysis within the nested case cohort study. Greater than 90% of the selected study children have available samples for analysis. Approximately 80% of the study participants have stool samples available for analysis. Adm = admission, disch = discharge.

Samples were collected at admission to hospital, discharge and at follow up timepoints. Standard operating procedures (SOPs) for collecting and handling biological samples were prepared and harmonized training was provided at each site to ensure comparability. Time from collection to freezing at -80°C was recorded for each sample with a target window of 30 minutes ( Figure 1). Sample types were stool, faecal swabs, whole blood, serum, plasma, and dry blood spots. For each type of sample, one or more aliquots were archived and no further aliquoting will be carried out before shipment. Samples collected have been transferred from sites to the KEMRI/Wellcome Trust Research Programme biorepository in Kilifi, Kenya.

***Stool*:** Faecal samples were collected in faecal pots at home or in hospital by the caregivers or study health workers and transported to the laboratory within 30 minutes in a temperature controlled cool box maintained at 2 – 8°C using ice packs. At the laboratory, samples were aliquoted into sterile, prelabelled and barcoded two mL cryovials and transferred into a -80°C freezer. No additives, preservatives or media was added to the faecal samples.

***Faecal swabs*:** Copan (Copan Diagnostics, USA) flocked rectal swabs were collected when study participants were lying on the left lateral position. The swab was inserted gently into the anal canal until it reached the stopper or until resistance was felt. The swab was rotated three times and then slowly removed and inserted into the barcoded retaining tube.

***Blood*:** Venepuncture and blood draw was done using aseptic techniques. For plasma, blood collected into EDTA tubes was centrifuged at ~100x g for ten minutes at 6°C and the sample divided into two aliquots and frozen at -80°C freezer. For serum, blood was collected into red top tubes and allowed to clot for ~30 minutes. Serum samples were obtained by spinning the clotted sample at 100x g for ten minutes at 4°C.

***Dried blood spot*:** A drop of 75 – 80 µL of whole blood was spotted on a barcoded Whatman Protein Saver 903 card (Merck, Germany), dried for 15 minutes and transferred into a zip lock bag with a desiccant and stored in a -80°C freezer.

The target turnaround time from sample collection to storage was ~30 minutes and a median of 44 minutes (interquartile range; IQR 35 – 57) was achieved ( Figure 1). All samples were collected and processed using aseptic techniques while maintaining cold chain from collection to storage. The median samples storage time was 2.2 years (IQR 1.8 – 2.7) before shipment for analysis.

**Figure 2.**
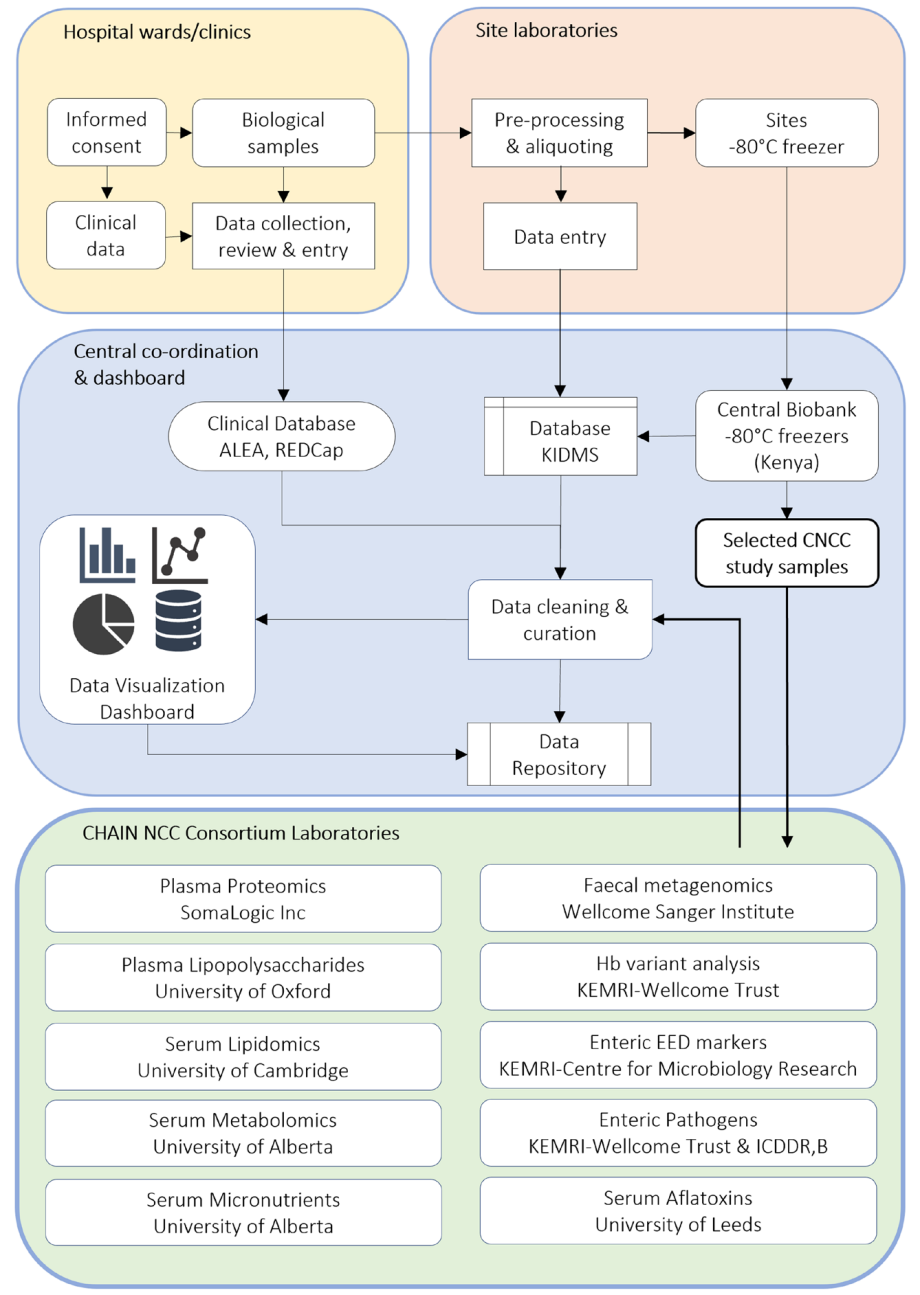
Samples collection, processing, storage and shipment workflow. Consent and sampling was carried out at the site’s ward or clinic. Samples were collected, pre-processed and stored at the study site -80 °C freezers. The duration from sampling to storage was ~44 minutes. Data curation and visualisation was done in R and the shiny application where queries and reports were transmitted online to sites for prompt and timed resolution. Data for samples was captured in the Kilifi Integrated Data Management System (KIDMS) that was linked to patient data in the clinical database captured on Research Electronic Data Capture (REDCap). Samples were subsequently shipped to the central biobank facility at KEMRI Wellcome Research Programme. CHAIN NCC study samples were analysed in the KEMRI-Wellcome Trust Research laboratories or shipped to consortium partners. CHAIN = Childhood Acute Illness and Nutrition, NCC = Nested Case Cohort, EED = Environmental Enteric Dysfunction, Hb = Haemoglobin, KEMRI-CMR = Kenya Medical Research Institute – Centre for Microbiology Research. TMIC = The Metabolomics Innovation Centre, ICDDR, B = International Centre for Diarrhoeal Disease Research, Bangladesh.

Samples were shipped from Kenya to consortium laboratories for analysis ( Figure 2). SOPs used at sites for sample collection, processing and storage can be downloaded at www.chainnetwork.org/resources.

### Study design

A nested case-cohort design drawing from a larger cohort provides estimates that are representative of the entire cohort while also yielding slightly more statistical power than a case control design ^59^. Case-cohort selection involves a random subsample of the original cohort (sub-cohort), independently of how cases are defined, followed by additional inclusion of all cases outside the sub-cohort creating the case-cohort set ^60^. This results in overrepresentation of the cases in the case-cohort set compared to the original cohort which is dealt with in analysis by weighting. A further advantage of this design over a case-control design is that the sub-cohort can also be used in analysis of other outcomes. In CHAIN, this could include outcomes of readmission or growth among survivors in future analyses, as well comparisons between acutely ill children and community children and between nutritional strata among acutely ill children in the current analysis.

**Figure 3.**
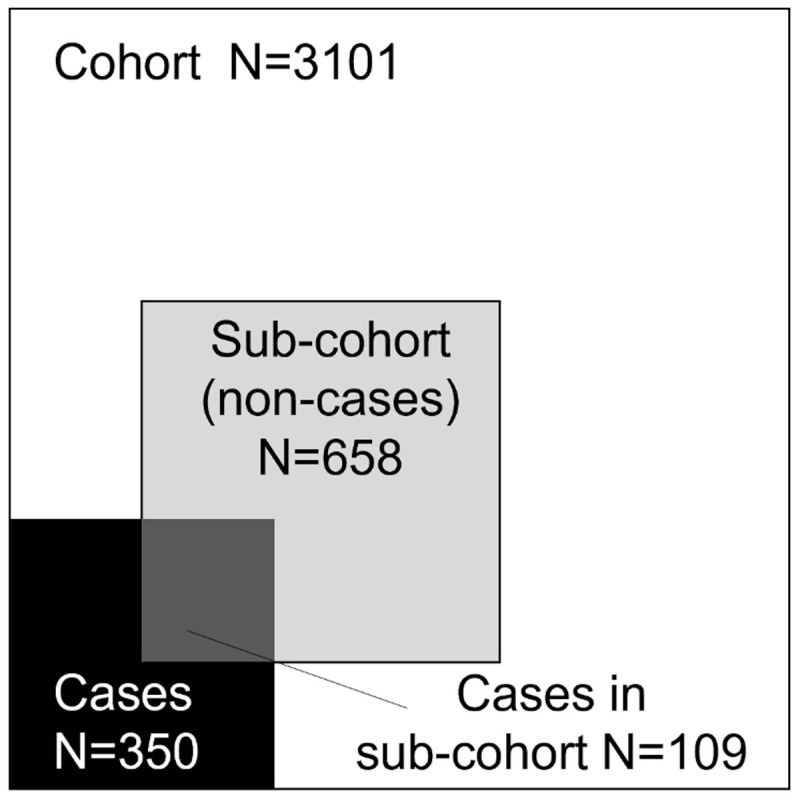
Sample selection for the CHAIN Nested Case-Cohort Study. Areas are proportional to the number of observations and excluding community participants are not included.

The original CHAIN cohort stratification was maintained in the CNCC design; thus, a random 24% sub-cohort of children enrolled in the CHAIN cohort stratified by site was selected. This included 658 survivors (sub-cohort non-cases) and 109 deaths (sub-cohort cases). Thereafter, all remaining deaths (n=241 cases) that were not selected in the random 24% sub-cohort ( Figure 3) were added, giving a total of 350 cases. An additional 30 randomly selected community participants from each site (total N=270) were chosen.

### Sample size

The primary analyses will be of acute mortality occurring within 30-days from admission and post-discharge mortality within 180-days from discharge using samples collected at admission and discharge timepoints. Power and effect size are a function of the number of cases, correlation between cases and survivors, and the ratio of cases to survivors. It is likely that the strength of association between exposures of interests and death will vary in magnitude between the 30-day and 180-day mortality periods. Sample size calculations suggest that a ratio of one case to two non-cases is adequate to reliably detect an HR of 1.5 at >80% power. The design selected all available deaths and simulation-based power calculations suggested that adding more non-cases per cases would yield minimal gains in power while incurring substantial additional expense.

### Laboratory analyses

The full laboratory protocol is described in the *detailed methods* section. Briefly, the aptamer based SomaScan assay will be used to quantify ~7000 proteins in plasma ^61^. We will also apply both targeted and untargeted mass spectrometry-based approaches to quantify metabolites including amino acids, acylcarnitines, biogenic amines, lipids, sugars, vitamins and minerals in serum ^62– 64^. Lipopolysaccharide levels in plasma will be measured via a limulus amoebocyte lysate-based, quantitative chromogenic endpoint assay ^65^. The gut microbiome will be determined through metagenomic sequencing of DNA extracted from stool samples. Further, faecal biomarkers of enteropathy including myeloperoxidase, alpha-1-antitrypsin, and calprotectin will be quantified in stool using ELISA. We will use quantitative polymerase chain TaqMan Array Card based assay ^66^ to test for a custom panel of 35 common enteric pathogens using nucleic acids extracted from rectal swabs. Finally, we will quantify haemoglobin variants using dried blood spots and aflatoxin-albumin ^67– 69^ in serum samples.

### Detailed methods

**Plasma proteomics:** The SomaScan assay that uses SOMAmer (Slow Off-rate Modified Aptamers) reagents to selectively bind and quantify proteins ^61^ will be used for plasma proteomics profiling. The SomaScan Assay quantitatively transforms the protein epitope availability in a biological sample into a specific SOMAmer-based DNA signal. A SOMAmer-protein binding step is followed by a series of partitioning and wash steps to convert relative epitope concentrations into measurable nucleic acid signals that are quantified using DNA-hybridisation microarrays. Briefly, diluted plasma samples are incubated with the respective SOMAmer reagent mixes that have been attached to streptavidin (SA)-coated beads (catch 1). Each of the SOMAmers is synthesized with photo-cleavable biotin, a spacer and cyanine-3 fluorophore at the 5’ end. The beads are washed to remove non-specifically associated proteins and other matrix constituents. Proteins that remain bound to SOMAmer reagents are labelled using an NHS-biotin reagent. SOMAmer complexes and unbound SOMAmer reagents are released from the SA beads using ultraviolet light that cleaves the photo-cleavable linker within the SOMAmer reagent construct into a solution containing an anionic competitor (biotin originally incorporated in the SOMAmer is left on the SA beads). Non-specific interactions, which have much faster dissociation rates compared to specific interaction, dissociate, and the polyanionic competitor solution prevents them from rebinding while specific complexes are maintained. The photo-cleavage eluate, which contains all SOMAmer reagents (some bound to a biotin-labelled protein and some free), is separated from the beads and then incubated with a second streptavidin coated bead that binds protein-SOMAmer complexes through biotin on the protein (catch 2). The inversion of polarity of attachment of protein-SOMAmer complexes on the second set of SA beads contributes a substantial degree of additional specificity enhancement by dramatically minimizing non-specific interactions due to multivalent interactions between proteins and SOMAmer-coated SA beads in the first (catch 1) incubation. The free SOMAmer reagents (which no longer contain biotin) are removed during subsequent washing steps. In the final elution step, protein-bound SOMAmer reagents are released from their proteins using denaturing conditions and recovered. These SOMAmer reagents are then quantified by hybridisation to custom DNA microarrays. The cyanine-3 signal from the SOMAmer reagent is detected on microarrays. The readout in relative fluorescent units (RFU) is directly proportional to the amount of target epitope in the initial sample. The resulting measurement and quantitation of ~7000 plasma proteins presented in a proprietary text-based format called ADAT. The *readat* R package which is free, open source, and available on Bioconductor and Bitbucket will be used for working with the SomaLogic ADAT files including importing, transforming and annotating data from these files ^70^.

**Serum Metabolomics:** We will apply a targeted quantitative metabolomics approach to analyse serum samples using a combination of direct injection mass spectrometry with a reverse-phase liquid chromatography tandem mass spectrometry (LC−MS/MS) custom assay. This custom assay, in combination with an ABSciex 4000 QTrap (Applied Biosystems/MDS Sciex, USA) mass spectrometer, can be used for the targeted identification and quantification of up to 150 different endogenous metabolites including amino acids, acylcarnitines, biogenic amines & derivatives, uremic toxins, glycerophospholipids, sphingolipids and sugars ^62, 63^. The method combines the derivatization and extraction of analytes, and the selective mass-spectrometric detection using multiple reaction monitoring (MRM) pairs. Isotope-labelled internal standards and other internal standards are used for metabolite quantification. The custom assay contains a 96 deep-well plate with a filter plate attached with sealing tape, and reagents and solvents used to prepare the plate assay. First 14 wells are used for one blank, three zero samples, seven standards and three quality control samples. For all metabolites except organic acid, samples are thawed on ice and vortexed and centrifuged at 13,000x g. Ten (10) µL of each sample is loaded onto the centre of the filter on the upper 96-well plate and dried in a stream of nitrogen. Subsequently, phenyl-isothiocyanate is added for derivatization. After incubation, the filter spots are dried again using an evaporator. Extraction of the metabolites is achieved by adding 300 µL of extraction solvent. The extracts are obtained by centrifugation into the lower 96-deep well plate, followed by a dilution step with MS running solvent.

For organic acid analysis, 150 µL of ice-cold methanol and 10 µL of isotope-labelled internal standard mixture are added to 50 µL of serum sample for overnight protein precipitation and subsequently centrifuged at 13000x g for 20 minutes. Fifty (50) µL of supernatant is loaded into the centre of wells of a 96-deep well plate, followed by the addition of 3-nitrophenylhydrazine reagent. After incubation for 2h, Butylated hydroxytoluene stabilizer and water are added before LC-MS injection.

Mass spectrometric analysis is performed on an ABSciex 4000 Qtrap® tandem mass spectrometry instrument (Applied Biosystems/MDS Analytical Technologies, Foster City, USA) equipped with an Agilent 1260 series UHPLC system (Agilent Technologies, Palo Alto, USA). The samples are delivered to the mass spectrometer by a LC method followed by a direct injection method. Data pre-processing is done using Analyst 1.6.2.

**Serum Micronutrients:** Both water-soluble and fat-soluble vitamins found in serum samples are analysed via targeted LC−MS/MS methods using previously described methods ^62^. To quantify the water-soluble vitamins in serum samples, 7-point calibration curves are generated by adding 10 μL of the isotopically labelled internal standard mixture to 50 μL of the calibration solutions in eppendorf tubes. Serum samples are also prepared by adding the isotopically labelled internal standard mixture to 50 μL of serum. A total of 60 μL of an aqueous Trichloroacetic acid solution (50 mg/mL) is pipetted to each eppendorf tube containing the calibrants or serum samples. Each tube is vortexed for 30 s for thorough mixing and then left on ice for 1 h. After cooling, each tube is centrifuged at 13 000 rpm for 20 min, and 100 μL of the supernatant transferred to a new HPLC vial. A volume of 10 μL is injected for LC−MS/MS analysis. An Agilent 1260 series UHPLC system (Agilent Technologies, Palo Alto, USA) coupled with an AB Sciex QTRAP 4000 mass spectrometer (Sciex Canada, Concord, Canada) is used to analyse water-soluble vitamins. An Agilent reversed-phase Zorbax Eclipse XDB C18 column (3.0 mm × 100 mm, 3.5 μm particle size, 80 Å pore size) coupled to a Phenomenex (Torrance, CA) SecurityGuard C18 precolumn (4.0 mm × 3.0 mm) is used for the separation of all water-soluble vitamins in the serum samples.

For the LC−MS/MS analysis of fat-soluble vitamins, 50 μL of calibration solutions or serum samples is pipetted into glass vials, followed by addition of 50 μL of internal standard mixture solution. Subsequently, 300 μL of methanol and 0.2 M ZnSO _4_ mixture solution (1:1 v/v) is added to precipitate the serum proteins and to facilitate the release of 25-hydroxyvitamin D3 from vitamin D binding protein. After this precipitation step, 1 mL of hexane is added to every sample to extract the fat-soluble vitamins. All the samples are vortexed for 10 minutes and centrifuged at 13 000 rpm for 20 minutes. Subsequently 650 μL of the hexane layer is then transferred to a new HPLC vial for evaporation under nitrogen gas at 40°C until dried. Finally, 200 μL of methanol is added to each dried sample to reconstitute the analytes, and 10 μL is injected for LC−MS/MS analysis. An Agilent 1260 series UHPLC system (Agilent Technologies, Palo Alto, CA) coupled with an AB Sciex QTRAP 4000 mass spectrometer (Sciex Canada, Concord, Canada) is used to analyse the fat-soluble vitamins in samples. A Phenomenex Kinetex C18 column (3.0 mm × 100 mm, 2.6 μm particle size, 100 Å pore size) connected to a Phenomenex SecurityGuard C18 precolumn (4.0 mm × 3.0 mm), is used to separate the fat-soluble vitamins. Data pre-processing is done using Sciex Analyst 1.6.2.

Metals and other trace elements in biological samples are measured using inductively coupled plasma mass spectrometry (ICP-MS) which is a powerful and sensitive approach. Trace elemental analysis is done using previously described methods ^62, 63^.

All metal analysis will be performed on a Perkin-Elmer NexION 350x ICP–MS (Perkin-Elmer, Woodbridge, ON, Canada), operating in a kinetic energy discrimination (KED) mode. Argon (ICP/MS grade, 99.99%) is used as a nebulizer (0.96 mL minute ^-1^), an auxiliary (1 mL minute ^-1^) and a plasma gas (16 mL minute ^-1^). Helium (He) is used as a non-reactive collision gas (Cell gas A: 4.3) to eliminate/minimize chemical interference. The dwell time for each metal ion is set to 50 ms with a total integration time of 500 ms (10 sweeps per reading and three replicates). The uptake of samples/standards/QCs is done by a peristaltic pump using the following protocol: (1) sample flush for 60 s at 48 rpm, (2) read delay for 15 s at 20 rpm, (3) spectral analyses at 20 rpm, and (4) washing for 60 s at 45 rpm. All samples are diluted using 1% HNO _3_, 5% H _2_O _2_, and MiliQ water (grade 1) by a factor of 10. Indium (In) is added to the dilution solvent as an internal standard. The final concentration of indium after mixing with the samples/standards is 20 ppb. An external calibration curve is used for the quantitation of all metal ions using a six- to seven-point calibration curve (for each metal) and linear regression. The performance of the ICP–MS is checked daily using a commercially prepared Perkin Elmer Nexion calibration solution to evaluate the sensitivity of the instrument. The Nexion solution is also used to calibrate the mass spectrometer at low (Be), mid (In), and high (U) masses. The accuracy of the ICP–MS analytical protocol will be evaluated in every sequence by the analysis of standard reference materials (SRMs)—i.e., serum and water QCs. Continuing calibration verification (CCV) is run every 15 samples to monitor the validity of each calibration curve throughout the sequence.

**Serum lipidomics:** For lipid profiling, the serum samples are extracted using the protein precipitation liquid extraction (PPLE) protocol and analysed by liquid chromatography with mass spectrometry detection (LC–MS) as described previously ^64^. All solvents and additives are of HPLC grade or higher and purchased from Sigma Aldrich (Haverhill, Suffolk, UK) unless otherwise stated. The PPLE protocol has been described previously ^64^. Briefly, 50 µL of plasma is transferred into a 2 mL screw cap Eppendorf plastic tube (Eppendorf, Stevenage, UK). Immediately, 650 µL of chloroform is added to each sample, followed by thorough mixing. Then, 100 µL of the LIPID-IS (5 µM in methanol), 100 µL of the CARNITINE-IS (5 µM in methanol) and 150 µL of methanol are added to each sample, followed by thorough mixing. Then, 400 µL of acetone is added to each sample. The samples are then vortexed and centrifuged for 10 minutes at 20,000 g to pellet any insoluble material. The supernatant is pipetted into separate 2 mL screw cap amber-glass auto-sampler vials (Agilent Technologies, Cheadle, United Kingdom). The organic extracts are dried down to dryness using a Concentrator Plus system (Eppendorf, Stevenage, UK) run for 60 minutes at 60°C. The samples are then reconstituted in 100 µL of 2: 1: 1 (propan-2-ol, acetonitrile and water, respectively) then thoroughly vortexed. The reconstituted sample is transferred to glass-coated 384-well plate (Esslab Plate+™), ready for LC-MS analysis.

Full chromatographic separation of intact lipids is achieved using Waters Acquite H-class UPLC System (Waters, Hertfordshire, United Kingdom) with the injection of 10 µL onto a Waters Acquity UPLC® CSH C18 column (Waters, Hertfordshire, United Kingdom); 1.7 µm, I.D. 2.1 mm × 50 mm, maintained at 55°C. Mobile phase A is 6:4, acetonitrile and water with 10 mM ammonium formate. Mobile phase B is 9:1, propan-2-ol and acetonitrile with 10 mM ammonium formate. The flow is maintained at 500 µL per minute through the following gradient: 0.00 minutes_40% mobile phase B; 0.40 minutes_43% mobile phase B; 0.45 minutes_50% mobile phase B; 2.40 minutes_54% mobile phase B; 2.45 minutes_70% mobile phase B; 7.00 minutes_99% mobile phase B; 8.00 minutes_99% mobile phase B; 8.3 minutes_40% mobile phase B; 10 minutes_40% mobile phase B. The sample injection needle is washed using 9:1, 2-propan-2-ol and acetonitrile. The Thermo Scientific Exactive Orbitrap mass spectrometer with a heated electrospray ionization source (Thermo Fisher Scientific, Hemel Hempstead, UK) is used. The mass spectrometer is calibrated immediately before sample analysis using positive and negative ionization calibration solution (recommended by Thermo Scientific). Additionally, the mass spectrometer scan rate is set at 4 Hz, giving a resolution of 25,000 (at 200 m/z) with a full-scan range of m/z 100 to 1,800 with continuous switching between positive and negative mode.

The instrument responses of the analytes, selection based on retention time and accurate *m/z*, are normalized to the relevant internal standard response (producing area ratios). These area ratios correct the intensity for any extraction and instrument variations. The area ratios are then blank corrected where intensities less than three times the blank samples are set to a ‘Not Found’ result (i.e., zero concentration). The accepted area ratios are then multiplied by the concentration of the internal standard to give the analyte semi-quantitative concentrations.

**Plasma lipopolysaccharides:** Endotoxin levels in EDTA plasma samples are measured via a Limulus Amoebocyte lysate-based, quantitative chromogenic endpoint assay (ThermoFisher, UK). The assay is performed based on a protocol described previously ^65^. Briefly, 50 µL of plasma is diluted 1:3 in 100 µL 10 mM MgCl _2_ solution (Lonza, Switzerland) to prevent inhibition of the Limulus Amoebocyte lysate assay by the EDTA present in the sample. The diluted samples are heated to 70°C for 30 minutes to inactivate interfering proteins, then further diluted 1:10 in 10 mM MgCl _2_ solution (Lonza, Switzerland), and plated (50 µL) in pyrogen-free 96-well plates for the Endotoxin detection assay. All samples are run in duplicates. The chromogenic endotoxin detection assay is conducted according to the manufacturer’s instructions. Absorbance is measured at 405 nm with the SPECTROstar Nano (BMG Labtech, Germany) against a standard curve of pure lipopolysaccharide (from *E. coli* O111:B4 (InVivogen, USA)) ranging from 0.05 EU/ml to 1.00 EU/ml. The detection range of the assay is 0.01 EU/ml to 1.00 EU/ml. Due to the variable baseline turbidity of plasma samples interfering with the absorbance measurement, a sample-specific blank is used alongside the standards. The absorbance of the sample-specific blank is deducted from the samples’ absorbance measurements before reading the values against the standard curve. Means of duplicate measurements are taken. Endotoxin concentrations are calculated from the standard curve using a linear model.

**Stool metagenomics:** Faecal samples in cryotubes are removed from the -80°C freezer, allowed to defrost and 30mg is used for DNA extraction. The FastDNA Spin Kit for Soil also incorporates a bead beating step to lyse bacterial cells which is performed using the FastPrep-24™ Classic bead beating grinder and lysis system (also supplied by MP Biomedicals). Negative controls using sterile Phosphate-buffered saline (PBS) are included for each extraction batch (24 samples per extraction batch). Total DNA is eluted in DNase/Pyrogen-free water and stored at -80°C until metagenomic sequencing. DNA samples, including negative controls, are quantified and subjected to paired-end (2 x 151bp) metagenomic sequencing on the NovaSeq (Illumina, USA) S4 XP with 192 samples per flow cell (targeting 13 million reads or 4 Gb / sample). Low-quality bases are trimmed using Trim Galore v0.5.0 with default parameters. Potential human contaminant sequences are removed by screening against the human genome (GRCh38) with BMTagger v1.1.0. Quality-controlled, paired-end reads will be independently assembled with metaSPAdes23 v3.13.0 using default parameters. Taxonomic classification from metagenomics reads will be performed using a reference genome mapping approach using the Human Gastrointestinal Bacteria Genome Collection enriched with Metagenome Assembled Genomes (MAGs) generated from the study metagenomes ^71^.

**Faecal pathogen detection:** Total nucleic acid will be extracted from rectal swab and tested using the enteric TaqMan Array Card (TAC, Life Technologies, USA) developed by the Houpt Lab at The University of Virginia ^66^. The custom enteric TAC targets multiple pathogens simultaneously including bacteria, viruses, helminths, and protozoa. The card is a 384-well microfluidic real-time PCR format for TAC-compatible instrument platforms. Briefly, both DNA and RNA are extracted from faecal swab specimens using the QIAamp Fast DNA Stool MiniKit (Qiagen, Germany) as previously described ^72^. The faecal sample undergo a lysate preparation process that includes mechanical disruption (bead beating), removal of inhibitors, purification and elution of DNA and RNA using spin columns. The extracted total nucleic acid is then used for testing. 40 µL of total nucleic acid extract is mixed with 50 µL of AgPath One Step RT-PCR buffer and 4 µL of enzyme mix (Life Technologies) in a 100 µL reaction, then loaded onto the microfluidic card by centrifugation at 1,200 rpm, 1 min for twice, followed by sealing and cutting off the loading ports. The cards are analysed in a ViiA7 or QuantStudio 7 Flex instrument (Life Technologies, USA). The cycling conditions are set as follows: 45°C for 20 minutes and 95°C for 10 minutes, followed by 40 cycles of 95°C for 15 s and 60°C for 1 minute. Extrinsic controls (MS2 bacteriophage and Phocine herpesvirus) are added to each sample during the lysate preparation to evaluate extraction and amplification efficiency while blank controls help rule out contamination during the extraction process. The assay uses predefined thresholds although the baseline for each target may require adjustment. A sample is deemed positive for a particular target when an appropriately shaped amplification curve crosses the analytical threshold for positivity before or at cycle 34.9 and negative if the curve does not cross the threshold line or crosses the threshold line at cycle 35.0 or later. Pathogen quantities in copy number are derived based on the standard curve for each target.

**Faecal biomarkers of enteropathy: Stool** myeloperoxidase, alpha-1-antitrypsin, and calprotectin will be quantified using the ELISA assay (Immundiagnostik AG, Germany) according to manufacturer’s instructions. The absolute concentration of the three biomarkers in samples will be calculated for 15 mg of stool using a dose response curve generated from the manufacturer’s standards.

**Haemoglobin variants:** Ion-exchange high-performance liquid chromatography (HPLC) will be used for separation and quantification of HB variants in dry blood spots (DBS) using the D-10 Haemoglobin Testing System (Bio-Rad, USA). DBS are resuspended on a lysis buffer to release haemoglobin. The samples are then diluted on the D-10 Haemoglobin Testing System (Bio-Rad, USA) and injected into the analytical cartridge. The D-10 delivers a programmed buffer gradient of increasing ionic strength to the cartridge, where the haemoglobin variants are separated based on their ionic interactions with the cartridge material. The separated haemoglobin then pass through the flow cell of the filter photometer, where changes in the absorbance at 415 nm are measured. The D-10 System will be used for quantitation of haemoglobins variants namely: A, S, F and A2 and other minor variants and diagnosis of ß-thalassaemia and sickle cell anaemia among study children.

**Serum aflatoxin:** The analysis of aflatoxin-albumin (AF-alb) will follow the method first described by Chapot and Wild ^73^ and detailed by Xu and colleagues ^67^. In brief, albumin is extracted from serum samples, hydrolysed overnight using pronase and purified with Sep-pak C-18 cartridges (Waters, MO, USA). AF-alb is quantified using a competitive ELISA ^67^ with a limit of detection (LOD) of 3pg AF-lysine equivalents per mg albumin. Each ELISA includes positive and negative control samples for quality control. All samples are measured in duplicate for each ELISA batch and repeated at least two times on separate days to confirm the results. Results are accepted when values within each ELISA have a %CV below 10% and samples tested on separate occasions had a %CV below 15%. Samples with concentrations above the linear range of the standard curve are repeated at a suitable dilution. This ELISA has been validated for measuring aflatoxin albumin biomarker levels against dietary aflatoxin intake ^68, 69^, and also against an LC-MS assay for measuring aflatoxin lysine ^74^. Whilst the ELISA consistently gives Af-alb values that are approximately three-fold higher than aflatoxin lysine values, the results between these methods correlate for the same samples. The AF-alb values are, therefore, suitable for use in analyses to compare variations in aflatoxin exposure in relation to health outcomes or other variables such as child growth.

### Statistical analysis

***Descriptive analysis.*** The distribution and prevalence of biomarkers in laboratory assays which have clear clinical interpretations will be described at admission and discharge timepoints across the nutritional strata and compared with community participants. These more targeted assays will include the serum micronutrients, serum aflatoxin biomakers, plasma lipopolysaccharides, haemoglobin variants, faecal biomarkers of enteropathy, and faecal pathogens. For these assays, the crude and confounder adjusted case fatality rates, which will be appropriately weighted for selection into the NCC analysis and the original CHAIN cohort, will be described.

The appropriate non-parametric statistical test will be employed to calculate the significance of the association between each feature and each outcome or measure of interest in every relevant subset of patients. For binary outcomes (i.e., the primary outcome of mortality), the Wilcoxon rank sum test will be used to determine the significance of the association while Hedge’s g will be used to calculate the effect size of the difference between groups. For continuous measures, the Spearman’s rank correlation coefficient will be used to assess the strength and significance of the association between the feature and the measure. Linear regression models will be fit for each feature against the confounding variables of interest and the residuals taken as the confounder-adjusted features.

### Integrated analysis

Advanced data integration and pathway analysis using systems biology data modelling to investigate integrated mechanistic pathways and reveal novel interactions between different biological modalities will be employed ^75^. Cross-validated multivariate models predicting the outcomes or measures of interest will be derived from the full dataset using a gradient-boosted tree algorithm (XGBoost) ^76^. A two-layer repeated cross-validation scheme will be used to prevent overfitting, optimize the parameters of the model, and get an estimate of the performance of the model on unseen patients. In brief, patients will be randomly and evenly split into a training set and a test set. An XGBoost model will be trained on the training set and then used to make predictions for the patients in the test set. This procedure will be performed 50 times using different and random train and test splits in each iteration. Final test predictions for each patient will be generated by integrating the model’s predictions for the patient using only the predictions from iterations in which the relevant patient was in the test set. Classifier model performance will be assessed with the area under the receiver operating characteristic curve (AUROC) and the area under the precision-recall curve (AUPRC) ^77^. Regression model performance will be assessed using Pearson’s correlation coefficient (Pearson’s R).

### Stacked generalization

To account for the differing information content between biological modalities and allow each modality to participate equally in the predictive models for the outcomes of interest, a stacked generalization approach will be employed ^75^. An independent predictive model will be trained using only the data from each modality while maintaining the same cross-validation folds across all modalities. A meta-learner will be subsequently trained to combine the predictions from each independent biological modality. Ultimately, each modality’s predictive model and the global meta-learner will be used to generate the final cross-validated test predictions for each patient in the dataset.

**Pathway enrichment analysis**

Gene set overrepresentation analysis of Gene Ontology (GO) Terms ^78^ for the top genes and proteins significantly associated with the outcomes of interest will be performed in R using the *topGO* package (Version 2.42.0) ^79^. The Biological Process ontology will be used to define GO terms for query. Fisher’s exact test will be used to calculate the *p-*value of enrichment for each GO term queried. Where relevant, top genes and proteins will be split into upregulated and downregulated sets before analysis.

### Adjustment for multiple comparisons

All *p*-values will be corrected for multiple hypothesis testing using Bonferroni’s correction where appropriate. In brief, the raw *p*-value will be multiplied by the total number of statistical tests performed to generate the adjusted *p*-value.

## Conclusions

The CHAIN Nested Case-Cohort Study is a unique opportunity to understand the mechanisms leading to early and later deaths among acutely ill children. The study brings together a consortium of multiple world-leading laboratories and integrated systems biology analyses of meta-dimensional data to provide a novel perspective on molecular mechanisms and converging pathological processes leading to mortality among acutely ill children. These data will inform the development of novel interventions aimed at mortality risk reduction among vulnerable children.

## Declarations

### Data availability

Where appropriate, the raw data will be deposited to public open access repositories. The processed data and analysis code will be deposited on the Harvard Dataverse under the Biosciences subtheme ( https://dataverse.harvard.edu/dataverse/kwtrp) and may be requested via email to dgc@kemri-wellcome.org.
