# Supplementary material for: The Childhood Acute Illness and Nutrition (CHAIN) network nested case-cohort study protocol: a multi-omics approach to understanding mortality among children in sub-Saharan Africa and South Asia
Source: Gates Open Res. 2022 Nov 3;6:77. Originally published 2022 Jul 18. [Version 2] doi: 10.12688/gatesopenres.13635.2 (PMC9646488; doi:10.12688/gatesopenres.13635.2)
Supplement: Peer review history

# Peer review: Reviewer response for version 2

**DOI:** 10.21956/gatesopenres.15370.r32750

**Published:** 2022-11-09

**Authors:** Quique Bassat

**Affiliations:** 1 Barcelona Institute for Global Health (ISGlobal), Hospital Clínic de Barcelona, University of Barcelona, Barcelona, Spain

I am satisfied with the responses to my comments, and happy for the paper to be published.

Is the study design appropriate for the research question?

Yes

Is the rationale for, and objectives of, the study clearly described?

Yes

Are sufficient details of the methods provided to allow replication by others?

Partly

Are the datasets clearly presented in a useable and accessible format?

Not applicable

Reviewer Expertise:

NA

I confirm that I have read this submission and believe that I have an appropriate level of expertise to confirm that it is of an acceptable scientific standard.

# Peer review: Reviewer response for version 1

**DOI:** 10.21956/gatesopenres.14914.r32265

**Published:** 2022-07-29

**Authors:** Quique Bassat

**Affiliations:** 1 Barcelona Institute for Global Health (ISGlobal), Hospital Clínic de Barcelona, University of Barcelona, Barcelona, Spain

This is a good manuscript reflecting rather accurately the protocol that the researchers have drafted for this nested case-control study. I have only a few comments to make which I believe would make the overall manuscript slightly clearer.

1. Abstract: "...270/1140 well community children". Would use "healthy" rather than "well".
2. Rationale/intro: Biological factors will surely have an enormous weight, but it is critical not to disregard "social" (i.e poverty-related; access-related, etc.) factors. The authors mention this, but it is not clear to me how deaths will be analysed taking them into account. This should be better explained. In other words, it may very well be that investigators find novel mechanistic hypotheses of mortality, but how much will those be put in the context of the overwhelming social determinants of death?
3. One of the missing pieces in this project is the link with the more thorough cause of death estimates. In other words, no postmortem work considered the gold standard approach for CoD investigation was part of or is planned as part of CHAIN, if I am not wrong. Doing all the mechanistic work without a clear description of the chain of events leading to death seems to me a bit of a futile exercise as investigators may be convinced that a given child died of disease X but may not have the clear postmortem confirmation of that supposition. How can this project link with already existing efforts (CHAMPS, etc.) in relation to CoD ascertainment and child mortality surveillance?
4. In sepsis (irrespective of underlying etioogy), one of the common pathways and transversal to different underlying infections is endothelial and immune activation, leading to loss of endothelial stability and eventual end-organ dysfunction. How many of these pathways are going to be investigated? How will their functionality be measured?
5. What will the methods be for CoD ascertainment in CHAIN? What methods have been used to investigate the underlying etiologies?
6. In Box 1, and understanding that you only discuss biological pathways, it may be useful to add a brief paragraph on their interrelation with social determinants.
7. Among the social data considered, is there any way of assessing socio-economic status? If so, which way?
8. Among the clinical data described, add "outcome data" and "Cause of Death" data, and specify by which methods they were determined, whether verbal autopsy, clinical records, etc.
9. I notice post-discharge period goes up to 6 months (180 days). How will you take into account mortality due to any other non-related causes (e.g. car accident) after discharge? Will you correct this? Wouldn't it make more sense to choose a shorter timeframe (i.e. 1 month or 3 months) rather than such a long period (6 months)?
10. I still find it unclear by reading the manuscript what the primary endpoints/measurements/objectives of this study are. Perhaps adding a final box at the end of the paper with "Expected results" may help it be more clear?

Is the study design appropriate for the research question?

Yes

Is the rationale for, and objectives of, the study clearly described?

Yes

Are sufficient details of the methods provided to allow replication by others?

Partly

Are the datasets clearly presented in a useable and accessible format?

Not applicable

Reviewer Expertise:

Infectious disease epidemiology, cause of death, mortality surveillance, malaria

I confirm that I have read this submission and believe that I have an appropriate level of expertise to confirm that it is of an acceptable scientific standard, however I have significant reservations, as outlined above.

# Response

**Published:** 2022-08-03

**Authors:** james njunge

**Reviewer: Quique Bassat**, Barcelona Institute for Global Health (ISGlobal), Hospital Clínic de Barcelona, University of Barcelona, Barcelona, Spain 

***This is a good manuscript reflecting rather accurately the protocol that the researchers have drafted for this nested case-control study. I have only a few comments to make which I believe would make the overall manuscript slightly clearer.***

***Abstract: "...270/1140 well community children". Would use "healthy" rather than "well".***

 Community children ^1^ were included in the study if they had no hospital admission in the 14 days prior to contact with the study team and were not ill and therefore are considered to be well and typical for the community rather than healthy. These community children were from the same neighbourhoods as acutely ill children and therefore, may have had anthropometric deficits, micronutrient deficiencies, helminths, etc. and so we would not regard them as optimally healthy.

***Rationale/intro: Biological factors will surely have an enormous weight, but it is critical not to disregard "social" (i.e poverty-related; access-related, etc.) factors. The authors mention this, but it is not clear to me how deaths will be analysed taking them into account. This should be better explained. In other words, it may very well be that investigators find novel mechanistic hypotheses of mortality, but how much will those be put in the context of the overwhelming social determinants of death?***

 The Childhood Acute Illness and Nutrition (CHAIN) cohort study aimed to characterise the biomedical and social risk factors for mortality in acutely ill children in hospitals and after discharge to identify targeted interventions to reduce mortality ^1^ (ref 58). Some of the biomedical and social factors which we examined that influence mortality among acutely ill children within 30 days of hospital admission and during the first 180 days following hospital discharge have already been described within the published CHAIN epidemiological paper ^2^ (ref 12). Briefly, lower nutritional strata at hospital admission, higher signs of illness severity at admission, HIV, underlying pre-existing medical conditions, adverse caregiver and household-level exposures, access to health care, and discharge against medical advice were associated with mortality. The specific focus of the CHAIN Network nested case-cohort (NCC) study is on generating mechanistic insights into the biological pathways leading to mortality, including those driven by nutritional and social factors.

***One of the missing pieces in this project is the link with the more thorough cause of death estimates. In other words, no postmortem work considered the gold standard approach for CoD investigation was part of or is planned as part of CHAIN, if I am not wrong. Doing all the mechanistic work without a clear description of the chain of events leading to death seems to me a bit of a futile exercise as investigators may be convinced that a given child died of disease X but may not have the clear postmortem confirmation of that supposition. How can this project link with already existing efforts (CHAMPS, etc.) in relation to CoD ascertainment and child mortality surveillance?***

 We would like to thank the reviewer for this comment. CHAIN ^2^ focussed on describing the upstream biomedical and social pathways underlying vulnerability since these are more amenable to interventions at a much earlier timepoint and not on the immediate causes of death which is the objective of the Child Health and Mortality Prevention Surveillance (CHAMPS) ^3^ Network. Unlike CHAMPS, CHAIN has considerable data in life in most cases long before the specific illness that caused death.

 Causes of death were allocated in CHAIN using the standard WHO methods to identify immediate and underlying causes of death, as presented in the published epidemiological paper ^2^. We recognize that such an approach has limitations.

***In sepsis (irrespective of underlying etiology), one of the common pathways and transversal to different underlying infections is endothelial and immune activation, leading to loss of endothelial stability and eventual end-organ dysfunction. How many of these pathways are going to be investigated? How will their functionality be measured?***

 Our previous work on mortality has shown that immune activation, endothelial dysfunction, and metabolic disturbances underlie mortality among acutely ill children with severe malnutrition ^4,5^. CHAIN NCC is combining data collected within the CHAIN cohort and conducting a large panel of omics laboratory investigations (systemic proteome, metabolome, lipidome, intestinal microbiome, etc.) to characterise multiple interconnected pathways including, but not limited to, endothelial function, immune function and inflammation, microbial translocation, micronutrient deficiency, altered metabolism and markers of organ dysfunction. We aim to use a systems biology data modelling approach to investigate these integrated mechanistic pathways and reveal novel interactions between different biological modalities.

***What will the methods be for CoD ascertainment in CHAIN? What methods have been used to investigate the underlying etiologies?***

 Details are provided in response to comment 4 above. Some underlying etiologies were determined during the cohort study, as presented in the published epidemiological paper ^2^. Others are being determined within the NCC, as described in this manuscript.

***In Box 1, and understanding that you only discuss biological pathways, it may be useful to add a brief paragraph on their interrelation with social determinants.***

 Details are provided in response 2 above. The focus of the CHAIN NCC is on generating mechanistic insights into the biological pathways leading to mortality with an overall aim of identifying novel biological interventions to reduce mortality among children in LMIC. The social determinants of death were described in the published epidemiological paper ^2^.

***Among the social data considered, is there any way of assessing socio-economic status? If so, which way?***

 This has already been described within the published epidemiological paper ^2^ with extensive details in the supplementary appendix.

***Among the clinical data described, add "outcome data" and "Cause of Death" data, and specify by which methods they were determined, whether verbal autopsy, clinical records, etc.***

 This has already been described within the published epidemiological paper ^2^

***I notice post-discharge period goes up to 6 months (180 days). How will you take into account mortality due to any other non-related causes (e.g. car accident) after discharge? Will you correct this? Wouldn't it make more sense to choose a shorter timeframe (i.e. 1 month or 3 months) rather than such a long period (6 months)?***

 Multiple studies in both low- and high-income settings indicated a prolonged increased mortality risk after admission (including >1 year) compared to the community peers ^6-8^ which is now a well-recognised phenomenon. This is one of the key puzzles that CHAIN is trying to unravel in LMIC settings. Of note, this may include a higher rate of traumatic deaths, possible because a child with impaired metabolic and immunological resilience or with underlying comorbidities may be more likely to die as a result of an injury than a child without these vulnerabilities.

***I still find it unclear by reading the manuscript what the primary endpoints/measurements/objectives of this study are. Perhaps adding a final box at the end of the paper with "Expected results" may help it be more clear?***

 We have indicated in the text under the sample size section that “ The primary analyses will be of acute mortality occurring within 30-days from admission and post-discharge mortality within 180-days from discharge using samples collected at admission and discharge timepoints.” Additionally, in the conclusion section, we have indicated that the analysis will “ provide a novel perspective on molecular mechanisms and converging pathological processes leading to mortality among acutely ill children. These data will inform the development of novel interventions aimed at mortality risk reduction among vulnerable children.”

**References**

 1. CHAIN. Childhood Acute Illness and Nutrition (CHAIN) Network: a protocol for a multi-site prospective cohort study to identify modifiable risk factors for mortality among acutely ill children in Africa and Asia. *BMJ open* **9**, e028454-e028454, doi:10.1136/bmjopen-2018-028454 (2019).

 2. CHAIN. Childhood mortality during and after acute illness in Africa and south Asia: a prospective cohort study. *The Lancet Global Health* **10**, e673-e684, doi:10.1016/S2214-109X(22)00118-8 (2022).

 3. Taylor, A. W. *et al.* Initial findings from a novel population-based child mortality surveillance approach: a descriptive study. *The Lancet Global Health* **8**, e909-e919, doi:10.1016/S2214-109X(20)30205-9 (2020).

 4. Wen, B. *et al.* Systemic inflammation and metabolic disturbances underlie inpatient mortality among ill children with severe malnutrition. *Science Advances* **8**, eabj6779, doi:doi:10.1126/sciadv.abj6779 (2022).

 5. Njunge, J. M. *et al.* Biomarkers of post-discharge mortality among children with complicated severe acute malnutrition. *Scientific Reports* **9**, 5981, doi:10.1038/s41598-019-42436-y (2019).

 6. Moisi, J. C. *et al.* Excess child mortality after discharge from hospital in Kilifi, Kenya: a retrospective cohort analysis. *Bulletin of the World Health Organization* **89**, 725-732, 732A, doi:10.2471/BLT.11.089235 (2011).

 7. Wang, H. E. *et al.* Long-term mortality after community-acquired sepsis: a longitudinal population-based cohort study. *BMJ Open* **4**, e004283, doi:10.1136/bmjopen-2013-004283 (2014).

 8. Winters, B. D. *et al.* Long-term mortality and quality of life in sepsis: a systematic review. *Crit Care Med* **38**, 1276-1283, doi:10.1097/CCM.0b013e3181d8cc1d (2010).

# Peer review: Reviewer response for version 1

**DOI:** 10.21956/gatesopenres.14914.r32267

**Published:** 2022-07-28

**Authors:** Michael Scott Freemark

**Affiliations:** 1 Division of Pediatric Endocrinology and Diabetes, Duke University School of Medicine, Durham, NC, USA

This should be a comprehensive analysis of the factors associated with mortality in malnourished children.

 Two questions:

1. I presume HIV status will be incorporated into the statistical analysis; is this correct?
2. The authors do not specify the measurement of inflammatory cytokines or hormones/growth factors shown previously to associate with mortality; is there a reason for that?

Is the study design appropriate for the research question?

Yes

Is the rationale for, and objectives of, the study clearly described?

Yes

Are sufficient details of the methods provided to allow replication by others?

Yes

Are the datasets clearly presented in a useable and accessible format?

Yes

Reviewer Expertise:

Pathogenesis of malnutrition, obesity and insulin resistance

I confirm that I have read this submission and believe that I have an appropriate level of expertise to confirm that it is of an acceptable scientific standard.

# Response

**Published:** 2022-08-03

**Authors:** james njunge

**Reviewer: Michael Scott Freemark**, Division of Pediatric Endocrinology and Diabetes, Duke University School of Medicine, Durham, NC, USA

***This should be a comprehensive analysis of the factors associated with mortality in malnourished children.***

***Two questions:***

***I presume HIV status will be incorporated into the statistical analysis; is this correct?***

 Yes, the HIV status will be included in the analysis. 

***The authors do not specify the measurement of inflammatory cytokines or hormones/growth factors shown previously to associate with mortality; is there a reason for that?***

 We will use the SomaScan platform that quantifies approximately 7000 proteins. The analytes detected include a large array of inflammatory proteins including cytokines and chemokines, hormones, and growth factors among others. See Plasma proteomics under the detailed methods section. The specific list of analytes will be provided in subsequent manuscripts.
